# Algorithms to predict cerebral malaria in murine models using the SHIRPA protocol

**DOI:** 10.1186/1475-2875-9-85

**Published:** 2010-03-24

**Authors:** Yuri C Martins, Guilherme L Werneck, Leonardo J Carvalho, Beatriz PT Silva, Bruno G Andrade, Tadeu M Souza, Diogo O Souza, Cláudio T Daniel-Ribeiro

**Affiliations:** 1 Laboratório de Pesquisas em Malária, Instituto Oswaldo Cruz, FIOCRUZ, Pavilhão Leonidas Deane sala 515 - Av. Brasil, 4365 - Manguinhos, Cep: 21045-900 - Rio de Janeiro - RJ, Brasil; 2 Departamento de Endemias Samuel Pessoa, Escola Nacional de Saúde Pública, FIOCRUZ, Rio de Janeiro, Brazil; 3 Departamento de Bioquímica, Universidade Federal do Rio Grande do Sul, Porto Alegre, Brazil; 4 La Jolla Bioengineering Institute, La Jolla, CA, USA

## Abstract

**Background:**

*Plasmodium berghei *ANKA infection in C57Bl/6 mice induces cerebral malaria (CM), which reproduces, to a large extent, the pathological features of human CM. However, experimental CM incidence is variable (50-100%) and the period of incidence may present a range as wide as 6-12 days post-infection. The poor predictability of which and when infected mice will develop CM can make it difficult to determine the causal relationship of early pathological changes and outcome. With the purpose of contributing to solving these problems, algorithms for CM prediction were built.

**Methods:**

Seventy-eight *P. berghei*-infected mice were daily evaluated using the primary SHIRPA protocol. Mice were classified as CM+ or CM- according to development of neurological signs on days 6-12 post-infection. Logistic regression was used to build predictive models for CM based on the results of SHIRPA tests and parasitaemia.

**Results:**

The overall CM incidence was 54% occurring on days 6-10. Some algorithms had a very good performance in predicting CM, with the area under the receiver operator characteristic (_au_ROC) curve ≥ 80% and positive predictive values (PV+) ≥ 95, and correctly predicted time of death due to CM between 24 and 72 hours before development of the neurological syndrome (_au_ROC = 77-93%; PV+ = 100% using high cut off values). Inclusion of parasitaemia data slightly improved algorithm performance.

**Conclusion:**

These algorithms work with data from a simple, inexpensive, reproducible and fast protocol. Most importantly, they can predict CM development very early, estimate time of death, and might be a valuable tool for research using CM murine models.

## Background

Cerebral malaria (CM) is one of the most important complications of *Plasmodium falciparum *infection. It is estimated that one in each four survivors may develop prolonged neurological and cognitive deficits [1]. CM is a multi-factorial disease with a poorly understood pathophysiology, but a number of host, parasite and epidemiological factors have been shown to play a role or influence pathogenesis [2-9]. Although no experimental model can fully reproduce all the aspects of the human disease, the murine models of CM have been instrumental in the study of CM physiopathogenesis, the most widely used model being the infection of different susceptible mouse strains by *Plasmodium berghei *ANKA [10], which shares many features with human CM [11]. *Plasmodium berghei *ANKA-infected susceptible mice develop a lethal neurological syndrome 6-12 days after infection with a cumulative incidence of 50-100% [12-14].

A number of approaches have been used to understand CM pathogenesis in the murine model, including interventional approaches, such as the blockage or addition of a given mediator [15], comparisons between CM-susceptible and resistant mouse strains [16,17] or between CM-inducing and non-inducing parasite strains [18,19]. Most approaches make it possible to demonstrate whether a factor is needed or not for the pathogenic process, but the precise role of each mediator and, mainly, a clear definition of the sequence of events during the course of the disease are difficult to determine. In particular, the association of physiopathological changes occurring early during infection with outcome, which is crucial for the understanding of CM pathogenesis, is curtailed by a number of factors related to the experimental model. First, the incidence of CM is variable among experiments, and can be as low as 50% [20]. Second, CM incidence can be spread over several days, most deaths occurring usually between days 6 and 9, but it can happen as early as day 5 and as late as day 12 of infection [12-14]. Therefore, the day of infection is usually a poor indicator of disease stage and, unless strict clinical assessment is performed, analyses based on the time of infection may give origin to bias derived from the different stages of the disease in different animals. Third, neurological signs (ataxia, convulsions, roll over, paralysis, coma) develop only few hours before death. Moreover, different host-parasite factors such as genetic background, age, inoculum size, course of parasitaemia and clonal variations of the parasite also interfere with the incidence of CM in mice [13,17,20]. These variations can, therefore, be a drawback for some experimental designs, for instance, in sequential studies aiming to identify early pathological changes related to CM development since, without a tool for predicting the outcome (development or not of the neurological syndrome), it may be complicated to determine which early changes are related to a given outcome. A way to solve or minimize this problem is to use factors or tests to build predictive models that can timely discriminate susceptible *P. berghei *ANKA-infected mice that will (CM+) or will not (CM-) develop CM.

It has been previously shown that a temperature below 30°C [21], the course of parasitaemia [22], and changes in locomotor activity [23] and in the TCR-β repertoire [10] are associated with death of *P. berghei *ANKA-infected mice. Lackner and colleagues [24], using a well-established protocol for behavioural evaluations in mice, the SHIRPA protocol, showed that CM+ mice present behavioural alterations strongly associated with a poor outcome 36 h prior to death. However, the study does not provide a tool for predicting CM development. The aim of the present study was to develop algorithms using logistic regression based on mouse behaviour data obtained with the SHIRPA primary screen and the levels of parasitaemia for early prediction of CM development in C57Bl/6 mice infected with *P. berghei *ANKA.

## Methods

### Mice

A total of 78 six- to eight-week-old female C57Bl/6 mice were obtained from CECAL/Fiocruz (Rio de Janeiro, Brazil) and used in five separate experiments. All mice were bred under specific pathogen-free (SPF) conditions and housed in groups of five (maximum), in plastic cages, with autoclaved food (Nuvital, Brazil) and filtered and autoclaved water ad libitum. All experimental protocols were reviewed and approved by the Fiocruz Ethics Committee on Animal Use (license number L-079/08).

### Parasite and infection

*Plasmodium berghei *ANKA strain was stored in Alsever's solution in liquid nitrogen until use. A sample was thawed and 100 **μ**L were injected intraperitoneally (i.p.) into a donor mouse of the same age as the experimental group mice. Three days later, blood was collected and 1 × 10^6 ^parasitized red blood cells (pRBC) were inoculated i.p. into each animal of the experimental group. Thin blood smears were daily made with a blood drop collected from the tip of the tail, stained according to the Panoptic method (Laborclin, Brazil) and examined under a light microscope (BH2, Olympus: Melville, New York, USA) with an oil immersion lens (1,000× final magnification). Parasitaemia levels were measured by counting the percentage of pRBC in at least 2,000 RBC. Mice were observed three times a day and CM diagnosis was made based on presentation of neurological clinical signs (ataxia, disorientation, paraplegia, roll-over and coma) on days 5 - 12 post-infection.

### Behavioural analyses

In order to provide a behavioural and functional profile of mice every 24 hours, from day 4 to day 10 of infection, SHIRPA primary screen was used as described elsewhere [25]. This protocol is composed of 40 separate parameters that provide a wide range of behavioural, neurological, and physiological measures. Briefly, each mouse was put under a clear cylinder with a diameter of 15 cm and 11 cm of height, which was located on a steel grid of about 1-cm mesh size. For 5 min, its behaviour was observed, the amount of rearing, grooming, and faecal pellets were counted and urination was scored as present or not. Subsequently, the mouse was transferred to a clear Perspex arena (55 × 33 cm) for evaluating the transfer arousal and observation of motor behaviour. Afterwards, a sequence of manipulations using tail suspension was performed and grip strength, body tone, and reflexes were recorded. To complete the assessment, the animal was restrained in a supine position to record autonomic behaviours prior to measurement of the righting reflex. Throughout this procedure, vocalization, urination, fear, irritability and aggression were recorded. All behaviours were scored to provide a quantitative assessment of each parameter as proposed by Hatcher *et al *[26]. Startle response, body length and temperature were not assessed. The values of the variables tremor, palpebral closure, piloerection, gait, positional passivity, wire manoeuvre, lacrimation, salivation, provoked biting, righting reflex, negative geotaxis, and fear were reversed to get a more reasonable physiological behaviour; i.e., the best and worst scores valued maximum and minimum points, respectively [24]. A detailed description of the tests and scoring system is provided as supplemental material (See Additional file 1: SHIRPA Protocol - *Primary screen*). A total score and scores measuring different neurological functions - reflex and sensory function, neuropsychiatric state, motor behaviour, autonomous function, and muscle tone and strength - were generated grouping individual scores obtained in each evaluation, as previously described [24,26]. However, different from Lackner *et al *[24], parameters were not normalized to a percentage of their respective maximum value before being summed up.

### Statistical analyses

Results of parasitaemia levels were expressed as means and standard errors of the mean (SEM). The initial statistical analyses of the SHIRPA individual scores were performed by nonparametric tests. Based on the scale of the individual parameter, Fisher's exact test (≤ 3 parameter values) or Wilcoxon rank-sum test (if > 3 parameter values) was used to compare CM+ and CM- mice in the same day of infection. Wilcoxon matched-pairs signed-ranks test was also used to evaluate the equality of matched pairs of observations from CM- mice on days 4 - 6 of infection. A p value of less than 0.05 was considered to indicate statistical significance. Kaplan-Meier curves were used to evaluate survival pattern of mice.

Multiple logistic regression analyses were used to identify which SHIRPA individual items were the most important prognostic factors for the occurrence of CM. In multiple logistic regressions, a forward stepwise selection procedure was performed using a p-value of 0.3 for the association with CM as the criterion for entering variables in the model. A backward stepwise elimination procedure was then performed, with a p-value of 0.05 as the criterion for remaining in the model.

**Figure 1:**
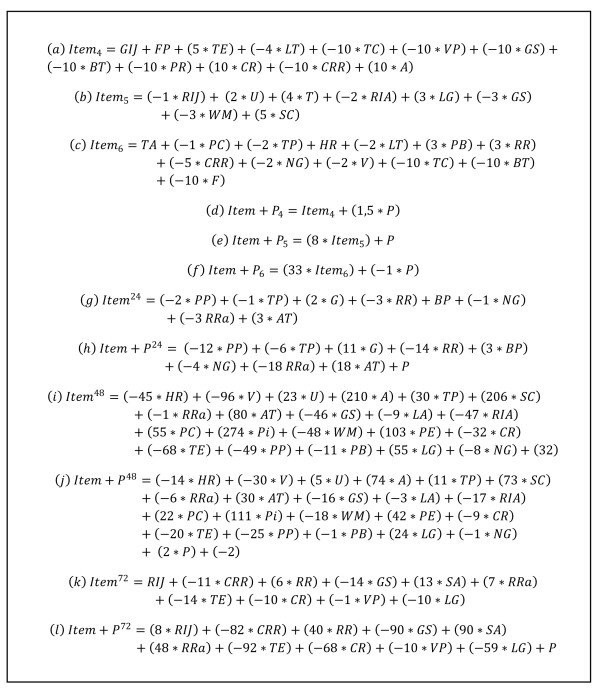
**Scores designed for prediction of CM using logistic regression**. Item and Item+P scores were designed for prediction of CM using a stepwise multiple logistic regression analysis to identify the most important prognostic factors of SHIRPA individual scores for the occurrence of CM. Item_x _and Item+P_x _scores are based on the day of infection and made to select animals that will develop CM. Item^y ^and Item+P^y ^scores were made to predict if mice will develop CM after 24, 48 or 72 hours. RIJ = Rearing in Jar; GIJ = Grooming in Jar; U = Urination; FP = Faecal Pellets; BP = Body Position; SA = Spontaneous Activity; RRa = Respiration Rate; T = Tremor; TA = Transfer arousal; LA = Locomotor Activity; RIA = Rearing in Arena; PC = Palpebral Closure; Pi = Piloerection; G = Gait; PE = Pelvic Elevation; TE = Tail Elevation; TEs = Touch Escape; PP = Positional Passivity; TC = Trunk Curl; LG = Limb Grasping; VP = Visual Placing; GS = Grip Strength; BT = Body Tone; PR = Pinna Reflex; CR = Corneal Reflex; TP = Toe Pinch; WM = Wire Manoeuvre; SC = Skin colour; HR = Heart Rate; LT = Limb Tone; AT = Abdominal Tone; L = Lacrimation; S = Salivation; PB = Provoked Biting; RR = Righting Reflex; CRR = Contact Righting Reflex; NG = Negative Geotaxis; F = Fear; I = Irritability; A = Aggression; V = Vocalization.

Predictive models based on a scoring system, with points allocated to each prognostic factor, were created from the final logistic regression model run in the test sample for days 4-6 of infection. The scoring system for each day was generated by dividing the value of the regression coefficient of each variable by the smallest coefficient and rounding the quotients to the closest integer [27]. As an example, tail elevation on day 4 had a regression coefficient of 1.115, which was then divided by the coefficient of faecal pellets (β = 0.2136), resulting in an approximate value of 5 (Item_4 _score, Figure 1a). The final score was obtained through the sum of each SHIRPA individual test score for those variables that remained in the final model multiplied by the coefficient generated. This approach resulted in the selection of individual SHIRPA tests that, when grouped, provided the best possible predictive power for CM development when applied on day 4 (Item_4_), day 5 (Item_5_) or day 6 (**I**tem_6_). For example, to obtain the final Item_4 _score for a mouse that had the following parameters on day 4: grooming in jar = 1; faecal pellets = 5; tail elevation = 3; limb tone = 1; trunk curl = 1; visual placing = 3_; _grip strength = 2_; _body tone = 1_; _pinna reflex = 1_; _corneal reflex = 1; contact righting reflex = 1; and aggression = 0_, _we used the formula shown in Figure 1a, as follows:

Item_4 _score = 1 + 5 + (3 _* _5) + (1 _* _(-4)) + (1 _* _(-10)) + (3 _* _(-10)) + (2 _* _(-10)) + (1 _* _(-10)) + (1 _* _(-10)) + (1 _* _(10)) + (1 _* _(-10)) + (0 _* _10), which would lead to a score of -63 points for a mouse presenting those characteristics.

In the case-control study, cases were considered as mice that died in a certain time following infection (e.g., day 6) and controls were those CM- mice at that time. This selection procedure was repeated for each day of infection in which CM+ mice died. Based on this sampling scheme, separate data sets were built for predicting death with CM 24, 48, 72 and 96 hours before. For example, in the "24 h-data set", the SHIRPA individual scores and parasitaemia levels 24 hours before death were recorded for cases and 24 h before that selected day for controls. The same procedure was used for the 48-, 72-, and 96-hour data sets. A new variable indicating the day of infection was created and was always considered in the models. Then, predictive scores were built in a similar way than those generated for each day of infection.

A cross-validation procedure was performed by randomly deleting one observation at a time, then predicting each removed individual from a model fit from the remaining data. This procedure was repeated many times and sensitivity and specificity of which were calculated with predictions from the models. The data that did not include the observations were used to fit the model. Cross-validated goodness-of-fit tests were also calculated [28]. The area under the receiver operator characteristic (_au_ROC) curve, sensitivity, specificity, and predictive values were used to evaluate models' performances. Predictive scores built in the case-control study were also evaluated in the original data set using the same cross-validation method to test models' performances in practice. All statistical analyses were performed using Stata 9.0.

## Results

### CM development

**Figure 2:**
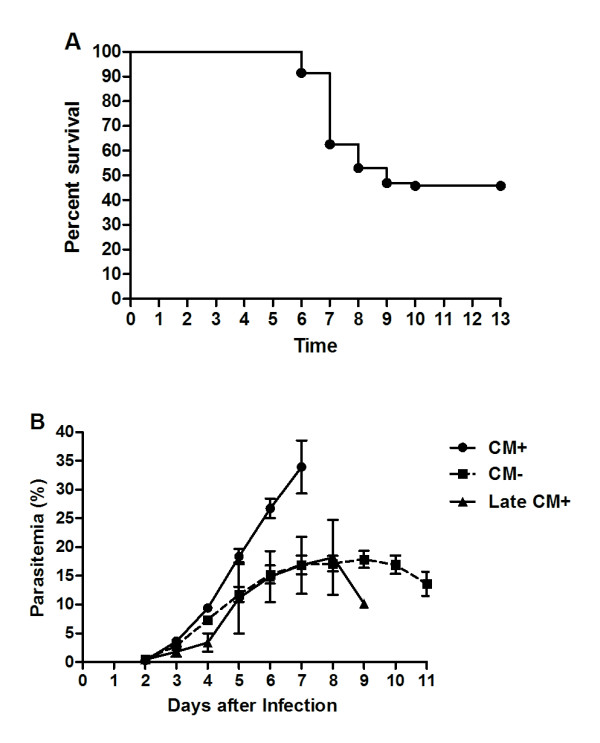
**Survival curve (A) and course of parasitaemia (B) of mice infected with *Plasmodium berghei *ANKA**. C57Bl/6 animals were infected with 1 × 10^6 ^*Plasmodium berghei *ANKA-parasitized red blood cells. CM+ = mice that developed cerebral malaria on days 6 - 8 after infection; CM- = mice that did not develop cerebral malaria; Late CM+ = mice that developed cerebral malaria on days 9 and 10 after infection. Results are expressed as means in (A) and means ± SEM in (B).

Inoculation of *P. berghei *ANKA to C57Bl/6 mice resulted in a lethal infection with a 100%-cumulative mortality on day 17. The cumulative incidence of CM was 54.3%, occurring on days 6 to 10 (Figure 2a). Mice that showed CM neurological signs (CM+ mice) died shortly thereafter, usually within a few hours. The remaining mice not affected by CM (CM- mice) died later on days 12-17 by other malaria-related pathologies (hyperparasitaemia, severe anemia) without showing any neurological sign.

### Course of parasitaemia

Parasitaemia was first detected 2 days after inoculation and grew rapidly for all infected mice up to day 4 of infection. As we have previously shown for other strains of mice [22], parasitaemia levels on subsequent days were critical for the outcome in terms of CM development. Indeed, parasitaemia levels in CM+ mice were significantly higher as compared to CM- mice on days 4 - 7 of infection (p < 0.05 in all time points, Figure 2b). Mice showing CM signs on days 9 - 10 had a different course of parasitaemia resembling CM- animals, being considered as "Late CM+" (Figure 2b).

Logistic regression models were used to assess whether parasitaemia levels on days 2-9 of infection predict the risk of CM development. Parasitaemia level of each mouse on days 4-7 was associated with the risk of CM development. Results from these logistic regression models on days 4-6 are shown in Table 1. On day 7, p-value was 0.015 and the odds ratio (OR) value was 1.068 (95% CI = 1.013-1.126). Parasitaemia level on other days was not associated with the risk of CM (p > 0.05).

**Table 1:** Association between parasitaemia level, total and functional category scores and the risk of CM development.

Score	Day 4			Day 5			Day 6		
			
	OR	p-value	95% CI	OR	p-value	95% CI	OR	p-value	95% CI
Parasitaemia	1.250	*0.048	1.002-1.560	1.080	*0.010	1.018-1.145	1.093	*0.001	1.039-1.151
Total	1.042	0.371	0.952-1.141	0.910	*0.003	0.855-0.969	0.907	* < 0.001	0.858-0.958
Reflex	0.978	0.941	0.548-1.746	0.928	0.682	0.650-1.325	0.491	* < 0.001	0.330-0.730
Neuro	0.981	0.923	0.669-1.438	0.719	*0.031	0.532-0.970	0.644	*0.001	0.495-0.839
Motor	1.000	0.998	0.892-1.122	0.833	*0.001	0.749-0.926	0.829	* < 0.001	0.747-0.920
Auto	1.186	0.299	0.859-1.638	0.932	0.393	0.793-1.095	0.883	0.185	0.735-1.061
Muscle	0.352	0.114	0.096-1.287	0.764	0.429	0.393-1.488	0.304	*0.001	0.148-0.621

Association between parasitaemia level, total and functional category scores based on the first screen of SHIRPA protocol on days 4-6 of infection and the risk of CM development in C57Bl/6 mice infected with *P. berghei *ANKA. OR = Odds Ratio; 95% CI = 95% Confidence Interval for odds ratio. *Denotes statistical significance in this table.

One example of the associative strength is the OR value for parasitaemia level observed on day 5 (OR = 1.080), indicating that an increase in the parasitaemia level by one unit raised the odds of CM development by 8% (Table 1). As CM+ mice started to show neurological signs and died on day 6, these data show that parasitaemia levels might be used to predict CM development at least 48 hours previous to death.

### SHIRPA assessment

To evaluate whether parameters from the SHIRPA primary screen were associated with CM development on days 4-6 of infection, individual scores obtained in each evaluation from CM+ mice were compared with those obtained from CM- mice on the same day (See Additional file 2: Supplementary Table). Total and functional category scores between CM+ and CM- mice were also compared. On day 4, none of the individual tests were significantly associated with CM development (p > 0.05 for all tests) and concordantly total and functional scores were also not associated (p > 0.05 for all scores). On day 5, nine tests were associated with CM (p < 0.05 for rearing in jar, locomotor activity, rearing in arena, touch escape, limb grasping, wire manoeuvre, lacrimation, righting reflex and vocalization). The majority of these tests measure motor behaviour (locomotor activity, limb grasping, and wire manoeuvre) and neuropsychiatric state (touch escape and vocalization), explaining the association found among these functional scores and CM (p < 0.001 and p = 0.027 respectively) on day 5. On day 6, twenty five tests were significantly associated with CM development (p < 0.05 for rearing in jar, grooming in jar, body position, tremor, transfer arousal, locomotor activity, rearing in arena, palpebral closure, gait, pelvic elevation, tail elevation, touch escape, trunk curl, limb grasping, body tone, pinna reflex, toe pinch, wire manoeuvre, skin colour, limb tone, provoked biting, righting reflex, contact righting reflex, negative geotaxis and vocalization). Again the majority of tests significantly associated with CM measure motor behaviour (10 tests), but all functional scores included tests significantly associated with CM on day 6 (reflex and sensory function - 3 tests; neuropsychiatric state - 3 tests; autonomous function - 2 tests; muscle tone and strength - 2 tests). On day 6, only the autonomous function score was not associated with CM (p > 0.05). Two tests carried out on day 5 (rearing in jar and rearing in arena) and four tests on day 6 (rearing in jar, grooming in jar, rearing in arena and contact righting reflex) were significantly associated with CM in spite of not being considered for the building of functional category and total scores by Hatcher *et al *[26].

All comparisons were made considering CM+ and CM- mice on the same day of infection because CM- mice performance in 11 tests significantly changed during the course of infection (faecal pellets, p = 0.025; visual placing, p = 0.046; grip strength, p = 0.015; corneal reflex, p = 0.025; toe pinch, p = 0.003; wire manoeuvre, p = 0.026; righting reflex, p = 0.026; rearing in jar, p = 0.017; urination, p = 0.045; spontaneous activity, p = 0.047; piloerection, p = 0.025).

Results from logistic regression models used to assess whether total and functional category scores on days 4-6 of infection predict the risk of CM development are shown in Table 1. Total, motor behaviour and neuropsychiatric state scores of each mouse on days 5 - 6 were associated with the risk of CM development showing that these scores can be used to predict CM development at least 24 hours previous to death. Reflex and sensory function and muscle tone and strength scores were also associated with CM, but only later on (day 6) (Table 1).

### Building and testing the predictive performance of scores

Since individual SHIRPA tests were not individually associated with CM development on day 4 of infection, different tests were combined in order to acquire predictive power. In addition, functional category scores indicate whether certain brain areas or functions are compromised in CM+ mice during the course of infection and are conceived to physiopathological purposes rather than to prediction. This could explain why these scores did not have a good predictive power when obtained on day 4 of infection. Therefore, new scores for days 4 - 6 were built assigning weights to each functional category score or making different combinations of specific SHIRPA tests (Item_x _scores, Figure 1) aiming at improving early prediction of CM. As parasitaemia levels showed to be an early but not very powerful predictor of CM, we also built scores combining parasitemia with functional and SHIRPA test scores (Item+P_x _scores, Figure 1). Generated scores with the highest performance for each day are shown in Figures 1a, b, c, d, e and 1f.

As hypothesized, _au_ROC curves increased when different weights were given to each functional score (from 38% to 66% on day 4, 72% to 78% on day 5 and 85% to 91% on day 6). _au_ROC curves increased when parasitaemia levels were considered in the model on day 4 and 5 (from 66% to 73% on day 4 and from 78% to 82% on day 5), but this phenomenon did not happen on day 6. When only the most predictive tests for each day together with parasitaemia levels were considered, _au_ROC curves increased even more (_au_ROC curves = 80%, 96% and 95% on days 4 - 6, respectively). As expected, _au_ROC curves increased with the proximity of the neurological syndrome manifestation, being more powerful on days 5 and 6 post-infection (72 - 96% and 85 - 95%, respectively) than on day 4 (38 - 80%). Although generated scores were generally more sensitive than specific, a relatively high prevalence of CM in the experiments may explain why it was observed positive predictive values (PV+) higher than negative predictive values (PV-) for most analysed scores. High PV+ values (≥ 92%) obtained for Item+P_x _scores on days 4-6 indicate that it is possible to early select mice with a high probability of developing CM.

### Case-control study

Although it is possible to early select CM+ mice using the Item+P_x _scores generated as described above, this kind of approach does not allow knowing on which day post-infection mice will develop CM. Susceptible mice can develop CM between day 6 and day 10, a long period of at least 120 hours, showing that CM+ mice selected by the generated scores are in different pathophysiological states. This can be a problem because some factors necessary or involved in CM pathogenesis could be present only in the middle or late in the course of infection, not being identified when the entire CM+ selected population was analysed. A way to solve this problem is to build scores to predict on which day mice will develop CM. To accomplish this, a case-control study controlled by the day of inoculation was designed using the same experiments performed for construction of the previous scores.

Scores were generated to predict whether a mouse would develop CM in 24, 48, 72 or 96 hours later. Again scores were generated assigning different weights to each functional category score or making different combinations of specific SHIRPA tests (Item^y ^scores) together or not with parasitaemia (Item+P^y ^scores). All generated scores for a 24 h-prediction showed an excellent performance (_au_ROC ≥ 84%) with the Item+P^24 ^score showing the best result (_au_ROC = 95%). Scores built for a 48 h-prediction had a good performance (_au_ROC between 78 - 94%) and the Item+P^48 ^score had an equivalent performance when compared to the Item+P^24 ^score (_au_ROC = 94%). Only Item+P^72 ^score had a good performance for a 72 h-prediction (_au_ROC = 85%), the remaining scores having only a fair predictive power (_au_ROC between 65 - 77%). All predictive scores for 96 h had a poor performance (_au_ROC ≤ 72%). Again, _au_ROC curves, sensitivity, and specificity increased with the proximity of the manifestation of the neurological signs, being more powerful within 24 hours of death. In general, specificity was greater than sensitivity for the generated scores, but a higher PV- obtained can be explained by the low prevalence of CM generated by the case-control study design. The high specificity (≥ 90%) of Item+P^24 ^and Item+P^48 ^scores indicates that they will possibly have higher PV+ when applied in practice and that they can be used to correctly classify CM+ mice according to the day of CM development. Generated scores with the highest performance are shown in Figures 1g, h, i, j, k and 1l.

### Algorithms to predict CM development

Whether Item+P_x _scores would be used in situations where a minimum error of 10% is acceptable, any of them could be chosen to correctly identify CM+ mice. However, for research purposes, an error of 10% is not acceptable in many situations. It is possible to solve this problem using a cut-off that improves specificity and PV+ values. These analyses are shown in Table 2. As hypothesized, increases in the cut-off values also increases specificity and PV+ until values ≥ 95% for all Item_x _and Item+P_x _scores.

**Table 2:** Best predictive performance of different logistic regression models.

Score	Day	_au_ROC	S	E	PV+	PV-	Acur.	n	Cut off	Score Value
Item+P_4_	4	79	68	89	95	47	73	38	80	-57
Item_5_	5	92	64	100	100	64	78	59	95	-5
Item+P_5_	5	96	81	100	100	77	88	59	75	-46
Item_6_	6	94	76	100	100	73	86	57	90	-37
Item+P_6_	6	95	77	100	100	73	86	57	90	-1244
Item^24^	5	77	25	100	100	95	95	61	40	-11
Item+P^24^	5	85	25	100	100	95	95	61	55	-37
Item^48^	6	91	14	100	100	89	89	56	85	250
Item+P^48^	6	93	14	100	100	89	89	56	80	123
Item^72^	4	82	46	100	100	77	80	37	65	-16
Item+P^72^	4	82	61	100	100	82	86	37	65	-83

Best predictive performance of different logistic regression models for correctly select mice that will develop CM in C57Bl/6 animals infected with P. berghei ANKA. Values are expressed in percents (%). auROC = area under the receiver operator characteristic; Se = sensitivity; Sp = specificity; PV+ = positive predictive value; PV- = negative predictive value; n = total of mice analysed; Cut-off = estimated probability of developing CM above which the score is considered positive; Score Value = score value corresponding to the respective cut off and above which a mouse can be considered positive in the score. Item_x _= Item scores designed for prediction of CM using the most important prognostic factors of SHIRPA individual scores on days 5 - 6 of infection; Item+P_x _= Item scores associated with parasitaemia on days 4 - 6; Item^y ^= Item scores for 24, 48, 72 or 96 hours prediction designed using the most important prognostic factors of SHIRPA individual scores; Item+P^y ^= Item scores associated with the levels of parasitaemia in the corresponding time.

**Figure 3:**
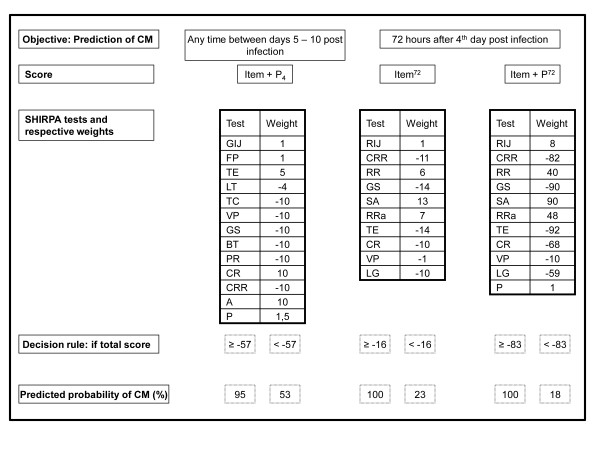
**A step by step protocol applied on day 4 post infection for predicting CM**. Item+P_4 _score should be used to estimate the probability of CM development on any time between days 5 - 10 post infection. Item^72 ^and Item+P^72 ^should be used to estimate the probability of CM development on day 7 post infection (72 hours after day 4 post infection). SHIRPA tests and its respective weights compounding each score are shown in the third line. Values obtained for each SHIRPA test should be multiplied by its respective weight and summed up to get a final score. A decision rule based on a specific final value for each score is shown. A final score above or equal (≥) this value is considered positive and is associated with a high probability of cerebral malaria (CM) development. On the other hand, a final score below this value (<) is considered negative and is associated with a low probability of CM. RIJ = Rearing in Jar; GIJ = Grooming in Jar; FP = Faecal Pellets; SA = Spontaneous Activity; RRa = Respiration Rate; TE = Tail Elevation; TC = Trunk Curl; LG = Limb Grasping; VP = Visual Placing; GS = Grip Strength; BT = Body Tone; PR = Pinna Reflex; CR = Corneal Reflex; LT = Limb Tone; RR = Righting Reflex; CRR = Contact Righting Reflex; A = Aggression; P = Parasitemia.

**Figure 4:**
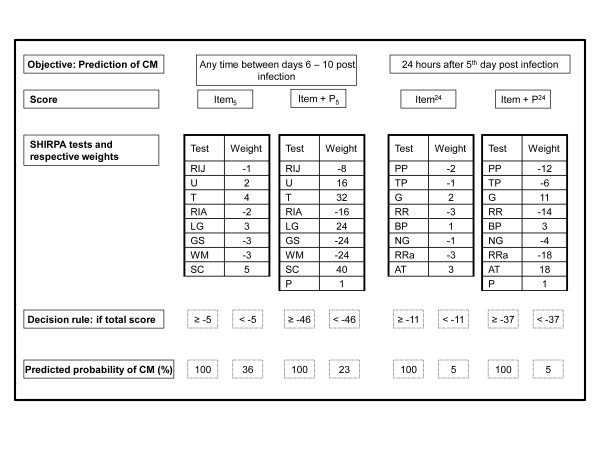
**A step by step protocol applied on day 5 post infection for predicting CM**. Item_5 _and Item+P_5 _scores should be used estimate the probability of CM development on any time between days 6 - 10 post infection. Item^24 ^and Item+P^24 ^should be used to estimate the probability of CM development on day 6 post infection (24 hours after day 5 post infection). SHIRPA tests and its respective weights compounding each score are shown in the third line. Values obtained for each SHIRPA test should be multiplied by its respective weight and summed up to get a final score. A final score above or equal (≥) this value is considered positive and is associated with a high probability of cerebral malaria (CM) development. On the other hand, a final score under this value (<) is considered negative and is associated with a low probability of CM. RIJ = Rearing in Jar; U = Urination; BP = Body Position; RRa = Respiration Rate; T = Tremor; RIA = Rearing in Arena; G = Gait; PP = Positional Passivity; TC = Trunk Curl; LG = Limb Grasping; GS = Grip Strength; TP = Toe Pinch; WM = Wire Manoeuvre; SC = Skin colour; AT = Abdominal Tone; RR = Righting Reflex; NG = Negative Geotaxis; P = Parasitemia.

**Figure 5:**
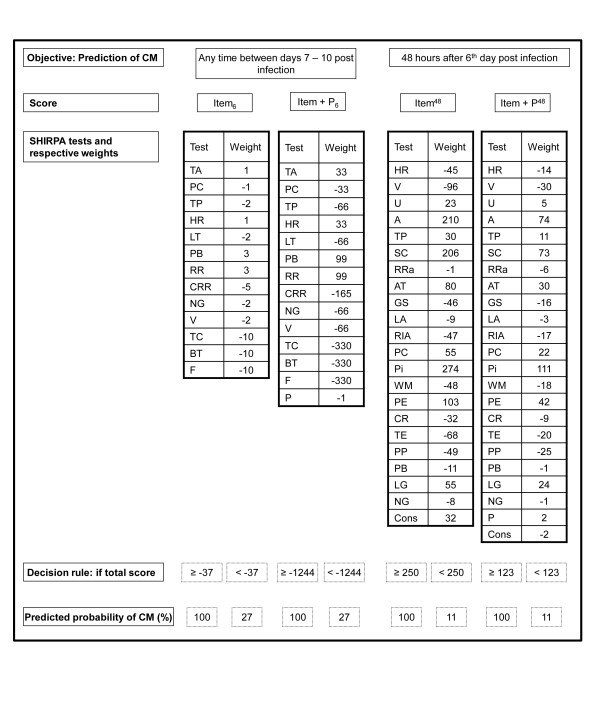
**A step by step protocol applied on day 6 post infection for predicting CM**. Item_6 _and Item+P_6 _scores should be used estimate the probability of CM development on any time between days 7 - 10 post infection. Item^48 ^and Item+P^48 ^should be used to estimate the probability of CM development on day 8 post infection (48 hours after day 6 post infection). SHIRPA tests and its respective weights compounding each score are shown in the third line. Values obtained for each SHIRPA test should be multiplied by its respective weight and summed up to get a final score. A final score above or equal (≥) this value is considered positive and is associated with a high probability of cerebral malaria (CM) development. On the other hand, a final score under this value (<) is considered negative and is associated with a low probability of CM. U = Urination; RRa = Respiration Rate; TA = Transfer arousal; LA = Locomotor Activity; RIA = Rearing in Arena; PC = Palpebral Closure; Pi = Piloerection; PE = Pelvic Elevation; TE = Tail Elevation; PP = Positional Passivity; TC = Trunk Curl; LG = Limb Grasping; GS = Grip Strength; BT = Body Tone; CR = Corneal Reflex; TP = Toe Pinch; WM = Wire Manoeuvre; SC = Skin colour; HR = Heart Rate; LT = Limb Tone; AT = Abdominal Tone; PB = Provoked Biting; RR = Righting Reflex; CRR = Contact Righting Reflex; NG = Negative Geotaxis; F = Fear; A = Aggression; V = Vocalization; Cons = constant; P = Parasitemia.

To verify Item^y ^and Item+P^y ^scores performance in practice and determine cut-offs that improve their specificities and PV+ values, these scores were cross-validated in the original data set used for the development of Item_x _and Item+P_x _scores. The performance of the best predictive scores on the best days is also shown in Table 2. In general _au_ROC curves were 5 - 20% worse on days 4 - 6 than when obtained in the case-control study. However, _au_ROC curves remained above 79% for Item+P^y ^and above 77% for Item^y ^scores on some specific days (Table 2). It was also possible to establish cut-offs with very high specificity and PV+ values for these scores on specific days (Table 2). These data show that at any time between days 4 and 6 it is possible to predict with high level of confidence which mice will develop CM (Figures 3, 4 and 5). Figures 3, 4 and 5 indeed provide researchers with a simple, fast and powerful screening/prediction tool that may prove useful in a wide range of applications. Furthermore, in some specific situations it is also possible to predict not only **if **but also when CM will occur: *i*) on day 4 it is possible to define which mice will develop CM on day 7 using Item^72 ^and Item+P^72^; *ii*) on day 5 it is possible to define which mice will develop CM on day 6 using Item^24 ^and Item+P^24^; *iii*) on day 6 it is possible to define which mice will develop CM on day 8 using Item^48 ^and Item+P^48 ^(Figures 3, 4 and 5). The cross-validation process revealed that combinations other than these do not provide high predictive powers.

Table 2 also show the score value that corresponds to the respective cut-off. This value represents the minimum value that a mouse must get in the respective score to be considered as a CM+ mouse. For example, a mouse with an Item+P_4 _score greater than -57 (-56, -55...) on day 4 will be considered as positive. For this cut-off point, the probability of CM development among those considered positive is 95% (Figure 3).

## Discussion

Previous studies showed that parameters as temperature and parasitaemia levels are associated with CM development and death in *P. berghei *ANKA-infected mice [21-24], but only one study from our group [22] measured the degree of this association (using hazard ratio) and none of them showed estimates of sensitivity and specificity for CM development. Collete *et al *[10] showed that it is possible to discriminate CM+ from CM- B10.D2 mice infected with *P. berghei *ANKA by analysing the TCRβ repertoire using a throughput CDR3 spectratyping method. However, for being sophisticated, expensive, and time-consuming, the use of this method for purposes of predicting CM does not seem promising.

The SHIRPA protocol is reproducible across time and laboratories [29,30] and it is a reliable tool for the evaluation of the neurological function in murine models of human disease [26,31-36]. Lackner *et al *[24] showed that many scores obtained from the SHIRPA primary screen are associated with CM development. However, the purpose of the study was to provide researchers with a tool for improved performance of clinical assessment of CM mice, not as a tool for predicting CM development. Our analyses confirm their data that motor behaviour and neuropsychiatric state functions are early compromised in the course of infection (day 5) and that scores measuring muscle tone and strength, reflex and sensory function are only compromised late in CM+ mice. On the other hand, the performance of CM- mice also varied in the course of infection at least in eleven tests on days 4-6, showing that these mice have some degree of neurological impairment during *P. berghei *ANKA infection. These data agree with findings from Carvalho *et al *[37] showing that, although CM incidence was 70% overall in their series, 100% of *P. berghei *ANKA-infected CBA mice analysed presented some degree of histological alteration in the brain on days 6-8 after infection; CM- mice had microhaemorrhages, although in a much lower frequency than CM+ animals.

Animal models are important in guiding the discovery of mechanisms of disease and potential targets for prophylactic or therapeutic interventions, and they are as well a critical step in testing new developed curative or preventive interventions [38]. This is not different for CM [15]. As predictive scores that correctly select CM+ mice allow the study of these animals before the development of CM, they have multiple applications in CM studies.

A major application for a predictive score for CM murine models is on studies of pathophysiology. A *sine qua non *condition to attribute causality is that the factor must precede the outcome it is assumed to affect, however temporal direction might be difficult to establish if initial forms of disease, as happen in CM, were difficult to observe [39]. As Item_x _and Item+P_x _scores can be used to correctly select CM+ mice before the development of CM on days 4 - 6, pathologic factors present in these mice that are not present in CM- mice have a high probability to be part of the causative mechanism of CM and not a consequence of it. Furthermore, Item^y ^and Item+P^y ^scores can also help to determine whether a given mediator is present 24, 48 or 72 hours before death in CM+ mice and whether the blockage or addition of this mediator at the corresponding time interfere with the outcome of the disease. This approach is useful to analyse the temporal sequence of appearance and time of exposition needed so that different factors can cause CM in this model. These predictive scores can also be a suitable tool for preventive therapy and treatment studies allowing the determination of how much time before death a drug or intervention can change the outcome of the disease.

To be applied in practice, a score should be obtained from a simple and fast process. Employing proposed scores in practice is simple and direct using the scores values showed in Table 2 and Figures 3, 4 and 5. In addition, it is possible to calculate it very fast using a computer algorithm. Per mouse, it takes up to 30 minutes to carry out the entire SHIRPA protocol and to calculate parasitaemia levels by microscopy. This time may be too long for many experimental approaches, making Pond and Pond+P scores not suitable for practical use. However Item scores are composed of 8 to 21 tests, taking between 6-11 minutes per mouse to be applied. Unfortunately, parasitaemia levels alone had a poor performance in predicting CM (_au_ROC < 72%). However, if we add this index to Item scores, time of testing increases but remains reasonable, and can still be shortened if other methods to estimate it, such as flow cytometry, are used [40,41]. In addition, the performance of Item scores was very good and similar to that of Item+P scores to select CM+ mice (Table 2), making Item scores applicable in practice.

## Conclusion

The present study provides consistent data supporting the idea that scores based on both the primary SHIRPA and from measuring parasitaemia levels are associated with and, then, can be used to predict the development of CM induced by a *P. berghei *ANKA infection in C57Bl/6 mice. The present study is the first to apply a commonly accepted behavioural protocol to build predictive scores of outcome in a well-established model of cerebral infectious disease.

As "Item" and "Item+P" scores are derived from a simple, highly reproducible, fast and cheap protocol, and since our results showed that they can reliably identify (or predict) CM+ mice and their time of death, we think that these scores might be a valuable tool to delineate the mechanisms underlying CM and for research with CM murine models. Neurophysiological, neuroanatomical, neurochemical and neuropharmacological research with CM murine models are some areas in which these scores can be applied.

## Competing interests

The authors declare that they have no competing interests.

## Authors' contributions

YCM carried out the SHIRPA experiments, participated in the statistical analysis and drafted the manuscript. GLW participated in the design of the study, performed the statistical analysis and helped to draft the manuscript. LJMC participated in the design of the study and helped to draft the manuscript. BPTS and BGA participated in the SHIRPA experiments. TMS trained YCM in the SHIRPA tests and helped to draft the manuscript. DOG participated in the design of the study and hosted some trainees in the SHIRPA tests in his laboratory. CTDR conceived of the study, and participated in its design and coordination and helped to draft the manuscript. All authors read and approved the final manuscript.

## Supplementary Material

Additional file 1 A protocol showing how the primary screen of SHIRPA tests were performed and scored. Click here for file

Additional file 2 Values of individual SHIRPA tests and functional category scores of infected non-CM (CM-) mice on days 4 - 6 post-infection were compared with cerebral malaria (CM+) mice in this Table. Click here for file
