# Supplementary material for: Algorithms to predict cerebral malaria in murine models using the SHIRPA protocol
Source: Malar J. 2010 Mar 24;9:85. doi: 10.1186/1475-2875-9-85 (PMC2850361; doi:10.1186/1475-2875-9-85)
Supplement: Additional file 1 — A protocol showing how the primary screen of SHIRPA tests were performed and scored. [file 1475-2875-9-85-S1.PDF]

SHIRPA Protocol - *Primary screen*

Behavioural Observation

This primary screen is based on that developed by Rogers *et al.* [25]. This standard method provides a behavioural and functional profile by observational assessment of mice. This test will indicate defects in gait or posture, motor control and co-ordination, changes in excitability and aggression, salivation, lacrimation, piloerection, defaecation, muscle tone and temperature. It also provides a gross measure of analgesia. All parameters are scored to provide a quantitative assessment which enables comparison of results both over time and between different laboratories.

The primary screen provides a behavioural observation profile and assessment of each animal begins by observing undisturbed behaviour in a viewing jar. In addition to the scored behaviours (Set 1), the observer looks for manifestation of bizarre or stereotyped behaviour and, convulsions, compulsive licking, self-destructive biting, retropulsion and indications of spatial disorientation are recorded. Thereafter, the mouse is transferred to the arena for testing of transfer arousal and observation of normal behaviour (Set 2). There follows a sequence of manipulations using tail suspension and the grid across the width of the arena (Set 3). To complete the assessment, the animal is restrained in a supine position to record autonomic behaviours prior to measurement of the righting reflex (Set 4). Throughout this procedure vocalization, urination and general fear, irritability or aggression are recorded.

Equipment

1. Clear perspex arena based on the dimensions of a rat cage (internal dimensions: 55 x 33 x18 cm). In the floor of the arena is a perspex sheet marked with 15 squares (11cm). A rigid horizontal wire (3 mm diameter) is secured across the rear right corner such that the animals cannot touch the sides during the wire manoeuvre. A grid (40 x 20 cm) with 12 mm mesh (approximate) is secured across the width of the box for measuring tail suspension and grip strength behaviour.
2. Two clear perspex cylinders (15 x 11 cm) which are used as viewing jars.
3. One grid floor (40 x 20 cm) with 12 mm mesh on which viewing jars stand.
4. Four cylindrical perspex supports (3 cm high x 2.5 cm diameter) to raise grids off bench.
5. One square (13 cm) stainless steel plate for transfer of animals to the arena.
6. A dowel rod with a fine stainless steel wire (15mm length and 0.15mm diameter) attached to end for measurement of pinna and corneal reflexes. The wire should be suitable for heat sterilisation. As an alternative, cut lengths of 3 / 0 Mersilk held in the forceps can be used.
7. A plastic dowel rod sharpened to a pencil point to test salivation and biting.
8. A pair of dissecting equipment forceps, curved with fine points (e.g. 125mm forceps, Philip Harris Scientific), for the toe pinch.

9. A stopwatch.
10. An IHR Click box is used for testing the Preyer and startle responses. The Click Box generates a brief 20KHz tone at 90dB SPL when held 30cm above the mouse. Contact Prof. K.P. Steel, MRC Institute of Hearing Research, University Park, Nottingham NG7 2RD.
11. A rectal thermometer is used to measure body temperature. The probe is 700mm long and approximately 1.5mm in diameter.
12. Scales to measure body weight.
13. A ruler.
14. A 30cm clear perspex tube for the contact righting reflex. The internal diameter should be 2.5-3.0cm (depending on the size of the mice to be tested as a snug fit is required).

Observation: in the original SHIRPA protocol, tremor, palpebral closure, piloerection, gait, positional passivity, wire manoeuvre, lacrimation, salivation, provoked biting, righting reflex, negative geotaxis, and fear have an inverted scoring system (higher scores for worse performances). The scoring system was modified (inverted) in these tests to allow proper performance of the statistical analyses.

Experimental Procedure

Set 1.

Behaviour recorded in the Viewing Jar

The animal is placed in the viewing jar for 5 minutes. This is located on top of a grid which is suspended above a piece of white paper. The following behaviours are recorded without disturbing the animal and the amount of urination or defaecation is monitored at the end of the observation period. Incidents of bizarre or stereotyped behaviour and convulsions are recorded separately.

1. Body Position (BP)

0= Completely flat
1= Lying on side
2= Lying prone
3= Sitting or standing
4= Rearing on hind legs
5= Repeated vertical leaping

2. Spontaneous Activity (SA)

0 = None, resting
1 = Casual scratch, groom, slow movement
2 = Vigorous scratch, groom, moderate movement
3 = Vigorous, rapid/dart movement
4 = Extremely vigorous, rapid/dart movement

3. Respiration Rate (RRa)

0 = Gasping, irregular
1 = Slow, shallow
2 = Normal
3 = Hyperventilation

4. Tremor (T)

0 = Important
1= Mild
2 = None

Set 2.

Behaviour recorded in the Arena.

A metal plate is inserted under the viewing jar and the animal transferred and briskly dropped onto the floor of the arena without being handled. The stop watch is started and the immediate reaction to the new environment is recorded.

1. Transfer arousal (TA)

0 = Coma
1 = Prolonged freeze, then slight movement
2 = Extended freeze, then moderate movement
3 = Brief freeze (few seconds), then active movement
4 = Momentary freeze, then swift movement
5 = No freeze, immediate movement
6 = Extremely excited ("manic")

2. Locomotor Activity (LA)

Number of squares entered by all four feet in 30 seconds.

3. Palpebral Closure (PC)

0 = Eyes closed
1 = Eyes 1/2 closed
2 = Eyes wide open

4. Piloerection (Pi)

0 = Coat stood on end
1 = None

5. Gait (G)

0 = Incapacity
1 = Limited movement only
2 = Fluid but abnormal
3 = Normal

6. Pelvic Elevation (PE)

0 = Markedly flattened
1 = Barely touches
2 = Normal (3mm elevation)
3 = Elevated (more than 3mm elevation)

7. Tail Elevation (TE)

During forward motion
0 = Dragging
1 = Horizontally extended
2 = Elevated / Straub Tail

8. Touch Escape (TEs)

Finger stroke from above.
0 = No response
1 = Mild (escape response to firm stroke)
2 = Moderate (rapid response to light stroke)
3 = Vigorous (escape response to approach)

9. Positional Passivity (PP)

Struggle response to sequential handling.

4 = Struggles when held by tail
3 = Struggles when held by neck (finger grip, not scruffed)
2 = Struggles when laid supine (on back)
1 = Struggles when held by hind legs
0 = No struggle

Set 3.

Behaviour recorded on or above the Arena

Remove the animal from the arena and grip tail between thumb and forefinger.

1. Trunk Curl (TC)

0 = Absent
1 = Present

2. Limb Grasping (LG)

0 = Absent
1 = Present

3. Visual Placing (VP)

Extension of forelimbs when animal lowered by base of tail from a height of approximately 15cm above wire grid.

0 = None
1 = Upon nose contact
2 = Upon vibrasse contact
3 = Before vibrasse contact (18mm)
4 = Early vigorous extension (25mm)

4. Grip Strength (GS)

Lower the animal and allow it to grip the grid then apply a gentle horizontal backwards pull.

0 = None
1 = Slight grip, semi-effective
2 = Moderate grip, effective

3 = Active grip, effective
4 = Unusually effective

5. Body Tone (BT)

Compress sides of animal between thumb and index finger.

0 = Flaccid, no return of cavity to normal
1 = Slight resistance
2 = Extreme resistance, board like

6. Pinna Reflex (PR)

Whilst animal is gently restrained on the grid touch the proximal part of the inner canthus lightly with the tip of the fine wire probe, look at ear retraction.

0 = None
1 = Active retraction, noderately brisk flick
2 = Hyperactive, repetitive flick

7. Corneal Reflex (CR)

Whilst the animal is gently restrained on the grid, touch the cornea lightly with the side of the fine wire probe, look at eye-blink response.

0 = None
1 = Active single eye blink
2 = Multiple eye blink

8. Toe Pinch (TP)

Gentle lateral compression of mid digit of hind foot with fine forceps. During this procedure the hind limbs are lifted clear of the grid.

0 = None
1 = Slight withdrawal
2 = Moderate withdrawal, not brisk
3 = Brisk, rapid withdrawal
4 = Very brisk repeated extension and flexion

9. Wire Manoeuvre (WM)

Animal is held above the wire by tail suspension and lowered to allow the forelimbs to grip the horizontal wire. The mouse is held in extension and rotated around to the horizontal and released.

0 = Falls immediately
1 = Unable to lift hindlegs, falls within seconds
2 = Unable to grasp with hindlegs
3 = Difficulty to grasp with hindlegs

4 = Active grip with hindlegs

Set 4.

Behaviour recorded during Supine Restraint

Animal is scruffed firmly and the following observed during supine restraint.

Measure body length in mm from tip of nose to base of tail.

1. Skin colour (SC)

Colour gradations of plantar surface and digits of forelimbs.
0 = Blanched
1 = Pink
2 = Bright, deep red flush

2. Heart Rate (HR)

Felt by palpation below sternum.
0 = Slow, bradycardia
1 = Normal
2 = Fast, tachycardia

3. Limb Tone (LT)

Resistance to gentle finger tip pressure on plantar surface of left right hind paw.
0 = No resistance
1 = Slight resistance
2 = Moderate resistance
3 = Marked resistance
4 = Extreme resistance

4. Abdominal Tone (AT)

Palpation of abdomen.
0 = Flaccid, no return of cavity to normal
1 = Slight resistance
2 = Extreme resistance, board like

5. Lacrimation (L)

0 = Present
1 = None

6. Salivation (S)

The dowel rod is gently inserted between the teeth at the side of the animal's mouth. Provoked biting and salivation are recorded.

The area of wetness in sub-maxillary area.

0 = Wet zone entire sub-maxillary area
1 = Slight margin of sub-maxillary area
2 = None

7. Provoked Biting (PB)

0 = Present
1 = Absent

8. Righting Reflex (RR)

The animal is held by the tail and flicked backwards through the air such that it performs a backward somersault when released. Observe the landing position.

0 = Fails to right when placed on back
1 = Lands on back
2 = Lands on side
3 = No impairment

9. Contact Righting Reflex (CRR)

Place the animal into the plastic tube and turn mouse upside down.
0 = Absent
1 = Present

10. Negative Geotaxis (NG)

Place the animal on horizontal grid. This is then raised to the vertical with the animal facing the floor. Start the stopwatch and observe for 30 seconds.

0 = Falls off
1 = Does not move within 30 seconds
2 = Moves, but fails to turn
3 = Turns but then freezes
4 = Turns and climbs the grid

11. Fear (F)

0 = Freezes during transfer arousal
1 = None

12. Irritability (I)

0 = None
1 = Struggle during supine restraint

13. Aggression (A)

0 = None
1 = Provoked biting or attack

14. Vocalization (V)

0 = None
1 = Provoked during handling
