# Supplementary material for: Algorithms to predict cerebral malaria in murine models using the SHIRPA protocol
Source: Malar J. 2010 Mar 24;9:85. doi: 10.1186/1475-2875-9-85 (PMC2850361; doi:10.1186/1475-2875-9-85)
Supplement: Additional file 2 — Values of individual SHIRPA tests and functional category scores of infected non-CM (CM-) mice on days 4 - 6 post-infection were compared with cerebral malaria (CM+) mice in this Table. [file 1475-2875-9-85-S2.DOC]

**Supplementary Table: Comparison of individual SHIRPA tests obtained from cerebral and non- cerebral malaria mice.**

| **Test** | **Day 4** | | | **Day 5** | | | **Day 6** | | |
| --- | --- | --- | --- | --- | --- | --- | --- | --- | --- |
|  | **CM-** | **CM+** | **p** | **CM-** | **CM+** | **p** | **CM-** | **CM+** | **p** |
| **Rearing in Jar** | 6 (3.8) | 7 (4.8) | NS | 1 (2.7) | 0 (1.3) | **0.026** | 1 (2.7) | 1 (2.0) | **0.035** |
| **Grooming in Jar** | 2 (1.2) | 2 (1.7) | NS | 2 (1.7) | 2 (1.7) | NS | 3 (2.9) | 1 (1.8) | **0.012** |
| **Urination** | 0 (0/0) | 0 (0/1) | NS | 1 (0/1) | 1 (0/1) | NS | 0 (0/1) | 1 (0/1) | NS |
| **Faecal Pellets** | 1 (1.3) | 2 (1.9) | NS | 1 (1.7) | 1 (1.2) | NS | 1 (0.9) | 1 (1.4) | NS |
| **Body Position** | 3 (3/3) | 3 (3/3) | NS | 3 (3/3) | 3 (3/3) | NS | 3 (3/3) | 3 (0/3) | **0.013** |
| **Spontaneous Activity** | 2 (1/2) | 2 (1/2) | NS | 1 (1/1) | 1 (1/1) | NS | 1 (1/1) | 1 (0/1) | NS |
| **Respiration Rate** | 2 (2/2) | 2 (2/2) | NS | 0 (0/2) | 0 (0/2) | NS | 0 (0/3) | 0 (0/2) | NS |
| **Tremor** | 2 (1/2) | 2 (1/2) | NS | 1 (1/1) | 1 (1/2) | NS | 1 (1/1) | 1 (1/2) | **0.010** |
| **Transfer arousal** | 3 (3/3) | 3 (3/3) | NS | 3 (1/3) | 2 (1/3) | NS | 2 (1/3) | 1 (1/2) | **0.004** |
| **Locomotor Activity** | 14 (6.8) | 15 (5.1) | NS | 9 (6.4) | 4 (4.3) | **0.001** | 6 (4.9) | 2 (3.2) | **<0.001** |
| **Rearing in Arena** | 1 (1.2) | 1 (2.0) | NS | 1 (1.2) | 0 (0.7) | **0.013** | 1 (0.9) | 1 (2.11) | **0.025** |
| **Palpebral Closure** | 2 (2/2) | 2 (2/2) | NS | 2 (2/2) | 2 (1/2) | NS | 2 (2/2) | 1 (1/2) | **<0.001** |
| **Piloerection** | 1 (1/1) | 1 (1/1) | NS | 0 (0/0) | 0 (0/0) | NS | 0 (0/0) | 0 (0/0) | NS |
| **Gait** | 3 (3/3) | 3 (3/3) | NS | 3 (3/3) | 3 (3/3) | NS | 3 (3/3) | 3 (1/3) | **0.049** |
| **Pelvic Elevation** | 2 (2/2) | 2 (2/2) | NS | 2 (2/2) | 2 (2/2) | NS | 2 (2/2) | 2 (0/2) | **0.022** |
| **Tail Elevation** | 1 (1/2) | 1 (1/2) | NS | 1 (1/1) | 1 (1/1) | NS | 1 (1/1) | 0 (0/0) | **<0.001** |
| **Touch Escape** | 2 (2/2) | 2 (2/2) | NS | 2 (1/2) | 1 (1/1) | **0.046** | 2 (1/2) | 0 (0/1) | **<0.001** |
| **Positional Passivity** | 4 (4/4) | 4 (4/4) | NS | 4 (4/4) | 4 (4/4) | NS | 4 (4/4) | 4 (2/4) | NS |
| **Trunk Curl** | 1 (1/1) | 1 (1/1) | NS | 1 (1/1) | 1 (1/1) | NS | 1 (1/1) | 1 (0/1) | **<0.001** |
| **Limb Grasping** | 1 (1/1) | 1 (0/1) | NS | 1 (0/1) | 0 (0/1) | **0.039** | 1 (0/1) | 0 (0/0) | **<0.001** |
| **Visual Placing** | 3 (3/3) | 3 (3/3) | NS | 3 (3/3) | 3 (3/3) | NS | 3 (3/3) | 3 (1/3) | NS |
| **Grip Strength** | 2 (2/2) | 2 (2/2) | NS | 2 (2/2) | 2 (2/2) | NS | 2 (2/2) | 2 (1/2) | NS |
| **Body Tone** | 1 (1/1) | 1 (1/1) | NS | 1 (1/1) | 1 (1/1) | NS | 1 (1/1) | 1 (0/1) | **<0.001** |
| **Pinna Reflex** | 1 (1/1) | 1 (1/1) | NS | 1 (1/1) | 1 (1/1) | NS | 1 (1/1) | 1 (1/1) | **0.024** |
| **Corneal Reflex** | 1 (1/1) | 1 (1/1) | NS | 1 (1/1) | 1 (1/1) | NS | 1 (1/2) | 1 (1/1) | NS |
| **Toe Pinch** | 2 (1/2) | 1 (0/2) | NS | 1 (1/2) | 2 (1/2) | NS | 2 (2/3) | 1 (1/2) | **<0.001** |
| **Wire Manoeuvre** | 4 (4/4) | 4 (3/4) | NS | 4 (4/4) | 3 (1/4) | **<0.001** | 3 (3/4) | 1 (1/3) | **<0.001** |
| **Skin colour** | 1 (1/1) | 1 (1/1) | NS | 1 (0/1) | 1 (1/1) | NS | 1 (1/1) | 1 (0/1) | **0.005** |
| **Heart Rate** | 1 (1/2) | 1 (1/2) | NS | 1 (1/2) | 1 (0/1) | NS | 1 (1/2) | 1 (0/1) | NS |
| **Limb Tone** | 2 (1/2) | 1 (1/2) | NS | 1 (1/2) | 1.5 (1/2) | NS | 2 (1/2) | 1(1/1) | **<0.001** |
| **Abdominal Tone** | 1 (1/1) | 1 (1/1) | NS | 1 (1/1) | 1 (1/1) | NS | 1 (1/1) | 1 (1/1) | NS |
| **Lacrimation** | 1 (1/1) | 1 (1/1) | NS | 1 (1/1) | 1 (1/1) | **0.018** | 1 (1/1) | 1 (1/1) | NS |
| **Salivation** | 2 (2/2) | 2 (2/2) | NS | 2 (2/2) | 2 (2/2) | NS | 2 (2/2) | 2 (2/2) | NS |
| **Provoked Biting** | 0 (0/0) | 0 (0/0) | NS | 0 (0/0) | 0 (0/0) | NS | 0 (0/0) | 1 (0/1) | **<0.001** |
| **Righting Reflex** | 3 (3/3) | 3 (3/3) | NS | 3 (3/3) | 3 (3/3) | **0.022** | 3 (3/3) | 2 (0/2) | **<0.001** |
| **Contact Righting Reflex** | 1 (1/1) | 1 (1/1) | NS | 1 (1/1) | 1 (1/1) | NS | 1 (1/1) | 0 (0/1) | **<0.001** |
| **Negative Geotaxis** | 4 (4/4) | 4 (4/4) | NS | 4 (4/4) | 4 (3/4) | NS | 4 (4/4) | 3 (0/4) | **<0.001** |
| **Fear** | 1 (1/1) | 1 (1/1) | NS | 1 (1/1) | 1 (1/1) | NS | 1 (1/1) | 1 (1/1) | NS |
| **Irritability** | 0 (0/1) | 0 (0/0) | NS | 0 (0/0) | 0 (0/0) | NS | 0 (0/0) | 0 (0/0) | NS |
| **Aggression** | 0 (0/0) | 0 (0/0) | NS | 0 (0/0) | 0 (0/0) | NS | 0 (0/0) | 0 (0/0) | NS |
| **Vocalization** | 1 (1/1) | 1 (1/1) | NS | 1 (1/1) | 0 (0/1) | **0.002** | 1 (0/1) | 0 (0/0) | **<0.001** |
| **Total** | 73 (10.9) | 76 (7.2) | NS | 66 (10.7) | 56 (9.9) | **<0.001** | 62 (12.0) | 40 (17.7) | **<0.001** |
| **Functional Categories:** |  |  |  |  |  |  |  |  |  |
| **Reflex** | 10 (1.4) | 10 (1.3) | NS | 10 (1.1) | 10 (1.7) | NS | 11 (2.0) | 7 (3.0) | **<0.001** |
| **Neuro** | 12 (2.1) | 12 (2.0) | NS | 11 (1.6) | 10 (1.9) | **0.027** | 11 (2.4) | 7 (3.4) | **<0.001** |
| **Motor** | 33 (7.3) | 34 (6.5) | NS | 28 (7.0) | 21 (5.7) | **<0.001** | 25 (6.5) | 13 (8.9) | **<0.001** |
| **Auto** | 11 (3.1) | 12 (2.2) | NS | 10 (3.3) | 9 (3.1) | NS | 9 (2.1) | 8 (3.4) | NS |
| **Muscle** | 6 (0.5) | 5 (0.6) | NS | 6 (0.9) | 7 (0.7) | NS | 5 (1.0) | 4 (1.6) | **<0.001** |

Values of individual SHIRPA tests and functional category scores of infected non-CM (CM-) mice on days 4 – 6 post-infection were compared with cerebral malaria (CM+) mice respectively. Data shown are median (upper/lower quartile) or mean (±SD) where appropriate. Analysis is by Fisher’s exact test (≤3 parameter values) or Wilcoxon rank-sum test (>3 parameter values). Significant analyses are highlighted with p-values shown. NS = not significant. Reflex = reflex and sensory function score; Neuro = neuropsychiatric state score; Motor = motor behavior score; Auto = autonomous function score; Muscle = muscle tone and strength score.
